# Adrenomedullin promotes intrahepatic cholangiocellular carcinoma metastasis and invasion by inducing epithelial-mesenchymal transition

**DOI:** 10.3892/or.2015.4034

**Published:** 2015-06-05

**Authors:** CHUANG ZHOU, YAN ZHENG, LIN LI, WENLONG ZHAI, RENFENG LI, ZHIWEN LIANG, LONGSHUAN ZHAO

**Affiliations:** 1 Department of General Surgery, The First Affiliated Hospital of Zhengzhou University, Zhengzhou, Henan 450052, P.R. China; 2 Institutes of Biomedical Science, Fudan University, Shanghai 200032, P.R. China; 3 Department of Dermatology, Zhengzhou Children's Hospital, Zhengzhou, Henan 450053, P.R. China

**Keywords:** adrenomedullin, epithelial-mesenchymal transition, intra-hepatic cholangiocellular carcinoma

## Abstract

Intrahepatic cholangiocellular carcinoma (ICC) is the second most common type of primary liver cancer. However, its etiology and molecular pathogenesis remain largely unknown. The present study aimed to investigate the association between adrenomedullin (ADM) and epithelial-mesenchymal transition (EMT) in ICC and to elucidate the underlying signaling pathway. We evaluated the clinical significance of ADM in 133 ICC patients using tissue microarray analysis of ICC tissues. We also investigated the mechanisms of ADM in ICC EMT-mediated metastasis in cholangiocarci-noma cell lines *in vitro*. The results revealed that ADM was upregulated in human ICC tissues (73/133) compared with that in healthy controls. ADM expression was positively correlated with shorter overall survival (P<0.01). The characteristics of EMT were induced *in vitro* by adenoviral transduction of ADM into HuCCT1 cells, resulting in the downregulation of E-cadherin and ZO-1, and the concomitant upregulation of N-cadherin and vimentin. Knockdown of ADM by short hairpin RNA in HUH28 cells expressing high levels of ADM was associated with the reversal of EMT. Functional studies revealed that ADM regulated the activation of ZEB1, which subsequently mediated EMT. The results of the present study suggest that ADM plays an important role in ICC metastasis, and that ADM signaling of EMT may represent a valuable therapeutic target in cancer patients.

## Introduction

Intrahepatic cholangiocarcinoma (ICC) is defined as a chol-angiocarcinoma located proximally to the second-degree bile ducts. It is an aggressive neoplasm associated with extremely poor survival. The incidence and mortality of ICC have increased worldwide over the past two decades (1–3). Surgical treatment is the only curative treatment option for ICC, and the 5-year survival rate for patients with unresectable ICC is currently <5% (4), compared with 20–44% in patients undergoing resection at early T1–T2 stages. Tumor recurrence and metastasis are common in ICC (5), and the prevention of recurrence is thus the key to improving patient overall survival (OS). Adrenomedullin (ADM) is a multifunctional regulatory and vasoactive peptide originally isolated from human pheo-chromocytoma (6). ADM overexpression has been detected in human breast, lung, ovarian, pancreatic, prostate and renal cancers (7–9). However, the physiologic significance of ADM in ICC metastasis and its underlying molecular mechanism are largely unknown. A better understanding of the biological characteristics of ICC that contribute to tumor invasion and metastasis is paramount for developing novel strategies to treat this cancer.

## Materials and methods

### Clinical samples and cell lines

This study included 80 men and 53 women with an average age of 55.6±12.8 years (range, 27–84 years), who had undergone curative resection at the First Affiliated Hospital of Zhengzhou University (Zhengzhou, Henan, China) from January 2005 to December 2008. The diagnosis of ICC was confirmed by morphological criteria, immunohistochemical (IHC) staining and clinical findings. None of the patients had received any preoperative anticancer treatment. The present study was approved by the Human Ethics Committee of the First Affiliated Hospital of Zhengzhou University, and informed consent was obtained from all patients according to the committee's regulations and the Declaration of Helsinki. The clinical characteristics of the patients with ICC are presented in Table I. Functional studies were performed using HuCCT1 and HUH28 cholangiocarci-noma cell lines (Shanghai Institutes for Biological Sciences, Chinese Academy of Sciences, Shanghai, China). The cell lines were cultured in RPMI-1640 medium supplemented with 10% fetal bovine serum (FBS), 100 U/ml penicillin, 0.1 mg/ml streptomycin and 2 mmol/l L-glutamine.

### Total RNA extraction, cDNA synthesis, RNA isolation and real-time polymerase chain reaction (RT-PCR)

Total RNA was extracted from ICC and liver tissues using TRIzol reagent (Invitrogen, Carlsbad, CA, USA). The quality and concentration of the isolated RNA were measured with a NanoDrop ND-1000 spectrophotometer (NanoDrop Technologies, Montchanin, DE, USA). First-strand cDNA was synthesized using both oligo-dT primers and random 6-mers and PrimeScript RT Enzyme Mix I according to the manufacturer's instructions (Takara, Otsu, Shiga, Japan).

RT-PCR was performed using specific TaqMan probes and primer sets to examine ADM RNA expression levels. TATA-box-binding protein (TBP), which is considered to be a reliable reference gene for quantitative PCR normalization in HCC specimens, was used as a control (10). Commercialized probes and primer sets specific for ADM and TBP were purchased from Applied Biosystems (Foster City, CA, USA). TaqMan primers and probes for ADM and TBP were designed using Primer Express (Applied Biosystems). The transcripts were amplified with the TaqMan One-Step RT-PCR Master Mix reagent and ABI Prism 7900HT sequence detection system (both from Applied Biosystems). The expression levels of the tested genes were quantified in relation to the expression of TBP using sequence detector software and the relative quantification method (Applied Biosystems). The relative ADM mRNA levels were determined using the 2^−ΔΔCT^ method (11).

### Tissue microarray (TMA) and IHC

A tissue microarray was constructed as described previously (12). Samples were taken from each representative tumor tissue and from liver tissue adjacent to the tumor (within 10 mm) to construct TMA slides (in collaboration with the Shanghai Biochip Company Ltd., Shanghai, China). Duplicate tissue cylinders were obtained from intratumoral and peritumoral areas (a total of 4 punches for each patient). Tissues were incubated with primary rabbit anti-ADM monoclonal antibody (1:200; Abcam, Cambridge, MA, USA), according to previously described IHC protocols (13), using the EnVision Plus detection system (EnVision; Dako, Carpinteria, CA, USA). Reaction products were visualized by incubation with 3,3-diaminobenzidine. Semi-quantitative analysis of IHC staining was performed by two experienced pathologists in two sections of each specimen in 10 fields from each section (magnification, ×200). Immunostaining scoring was based on the intensity of staining and the percentage of positively stained cells: negative (−), 0–5%; intermediate (+), >5–10%; moderate (++), >10–25%; strong (+++), >25%. ADM staining ≥5% was considered positive.

### Construction of recombinant plasmids and transfection

Full-length human ADM cDNA was amplified by PCR and cloned into the pEGFP-N1 expression vector (Clontech, Palo Alto, CA, USA) to construct pEGFP-N1-ADM, and then transfected into HuCCT1 cells using Lipofectamine 2000 (Invitrogen) according to the manufacturer's instructions. Cells transfected with pEGFP-N1 were used as a negative control. Stable ADM-expressing clones were selected using geneticin (Roche Diagnostics, Indianapolis, IN, USA) at a concentration of 500 *µ*g/ml.

### Establishment of ADM-knockdown cells

Lentivirus containing short hairpin RNAs targeting ADM was purchased from GeneCopoeia (Rockville, MD, USA) and transfected into HUH28 cells using Lipofectamine 2000 (Invitrogen) according to the manufacturer's instructions. Cells transfected with the empty vector were used as controls. Stable clones were selected using puromycin (final concentration, 2 *µ*g/ml).

### In vitro cell behavior assay

Cellular proliferation was assayed in cells seeded at a density of 5×10^3^ cells/well in 96-well plates. The proliferation of the transfected cells was measured using a CyQUANT Cell Proliferation Assay kit (Invitrogen). Each assay was repeated 3 times. For wound-healing assays, monolayers of cells were wounded by scraping with a plastic pipette tip followed by rinsing several times with medium to remove dislodged cells. Cells that had migrated into the wound area were photographed. For invasion assays, 2×10^5^ cells were plated into the upper chamber of a polycarbonate Transwell filter chamber coated with Matrigel (BD Pharmingen, San Diego, CA, USA) and incubated for 48 h. The cells that migrated to the underside of the membrane were stained with Giemsa (Sigma-Aldrich, St. Louis, MO, USA) and counted under a microscope (Olympus, Japan).

### Statistical analysis

Survival was analyzed using the Kaplan-Meier estimate, and values were compared using the log-rank test. Independent prognostic factors were identified by multivariate survival analysis using a Cox proportional hazards model. The median value was used to determine the cut-off value for high vs. low expression of ADM. Statistical analyses were performed with SPSS 16.0 for Windows (SPSS software; SPSS Inc., Chicago, IL, USA). Statistical significance was accepted for P<0.05 for all tests.

## Results

### Expression of ADM in human ICC

**Figure 1:**
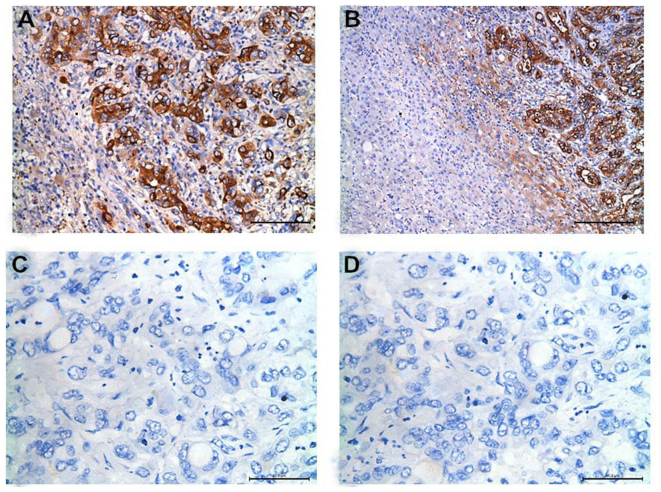
Adrenomedullin immunohistochemical staining of tumor tissue. The specimens from ICC patients were evaluated by immunohistochemistry using a manual quantitative-scoring method derived from staining intensity and extensity. Shown are representative samples with a high (A and B) and a low (C and D) score.

**Figure 2:**
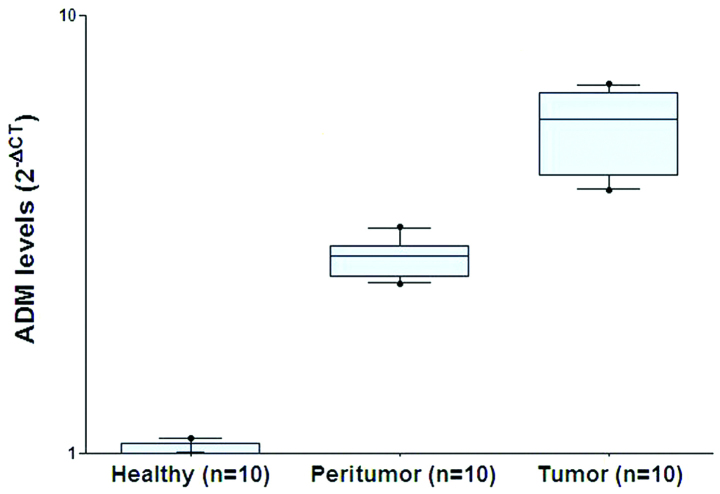
ADM PCR levels in the ICC patients and controls. ADM levels in ICC patient tumor specimens were compared with that of healthy control groups and peritumor specimens. ADM, adrenomedullin.

The significance of ADM expression in ICC patients was investigated by IHC (Fig. 1). ADM staining was mainly located in the cytoplasm of tumor cells. Most stromal cells were negative for ADM. As shown in Fig. 2, ADM mRNA expression was significantly higher in the ICC tumor tissues compared with that in the peritumoral and healthy liver tissues, according to RT-PCR.

**Figure 3:**
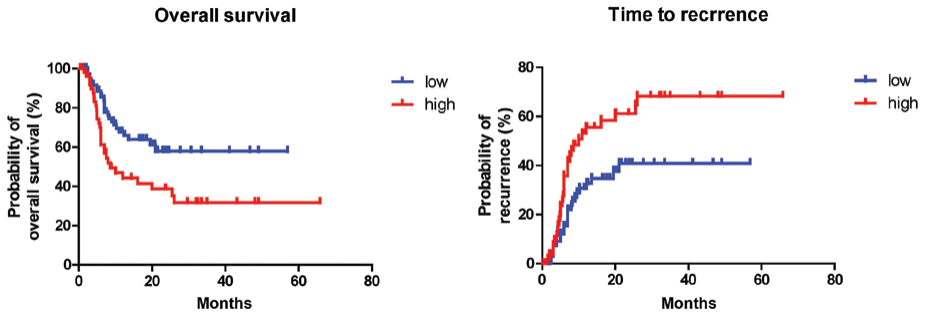
Kaplan-Meier analyses of the association of the ADM levels with OS and TTR for patients with ICC. The high and low groups were plotted according to the cut-off value defined as the median ADM level of the cohort. ADM, adrenomedullin; TTR, time to recurrence; OS, overall survival.

We investigated the clinical significance of ADM overex-pression in ICC by tissue microarray analysis of ICC tissues from 133 patients who underwent resection (Table I). Kaplan-Meier analysis revealed that patients with high ADM expression had poorer OS and shorter time to recurrence (TTR) (both P<0.01, Fig. 3). Univariate Cox regression analysis identified differentiation, encapsulation and ADM overexpression as factors significantly associated with OS, while multivariate Cox proportional hazards regression analysis identified differentiation and ADM overexpression as independent prognostic factors for OS in ICC patients (Table II).

### Effect of ADM on ICC cell growth

**Figure 4:**
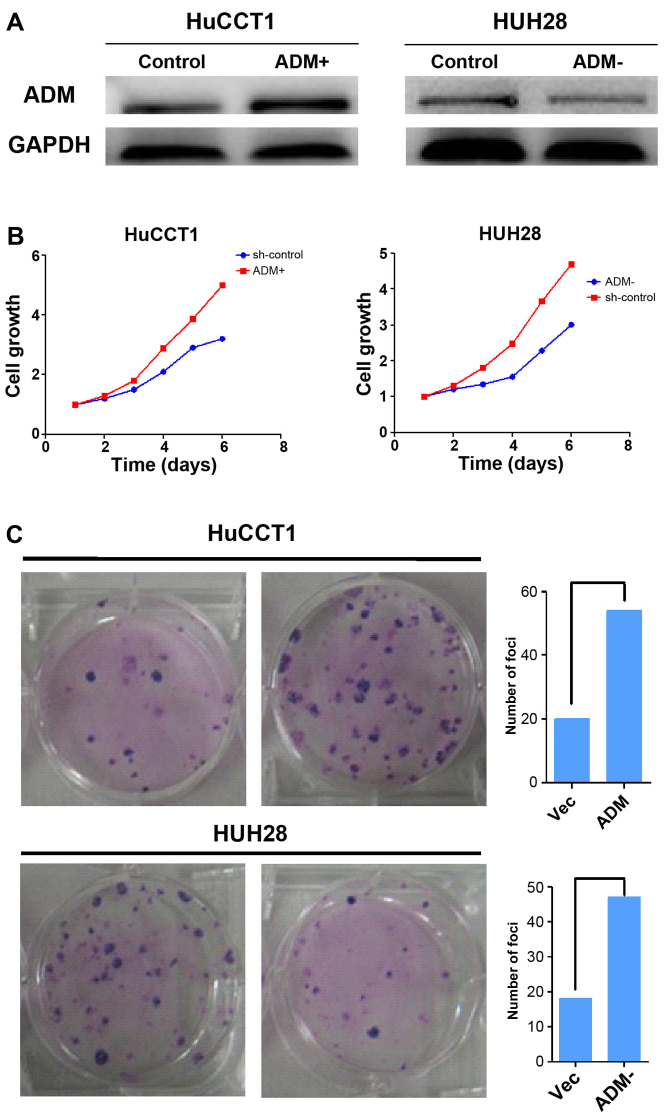
(A) Western blot analysis showing ectopic expression and silencing of ADM in the ADM-transfected cells. Expression of GAPDH was used as a loading control. (B) Rate of cell growth between ADM- and empty vector-transfected cells by XTT assay. The results are expressed as the mean ± SD of three independent experiments (^**^P<0.01, independent Student's t-test). (C) Representative images of increased foci formation in monolayer culture induced by ADM expression and silencing. Quantitative analyses of foci numbers are shown in the right panel. Values are reflected as the mean ± SD of at least three independent experiments (^**^P<0.01, independent Student's t-test). ADM, adrenomedullin.

To confirm the involvement of ADM in the growth of ICC cells, we transfected HuCCT1 cells with ADM and silenced HUH28 cells with short hairpin RNA, respectively. ADM expression was determined by western blotting (Fig. 4A). The tumorigenicity of ADM was determined by functional assays. The cell growth rates in the ADM-transfected cells were significantly higher than the rates in the control cells (P<0.01, Fig. 4B). ADM-transfected cells also formed significantly more and larger colonies (P<0.01) than the control cells (Fig. 4C).

### Effects of ADM on ICC cell migration and tumor metastasis

**Figure 5:**
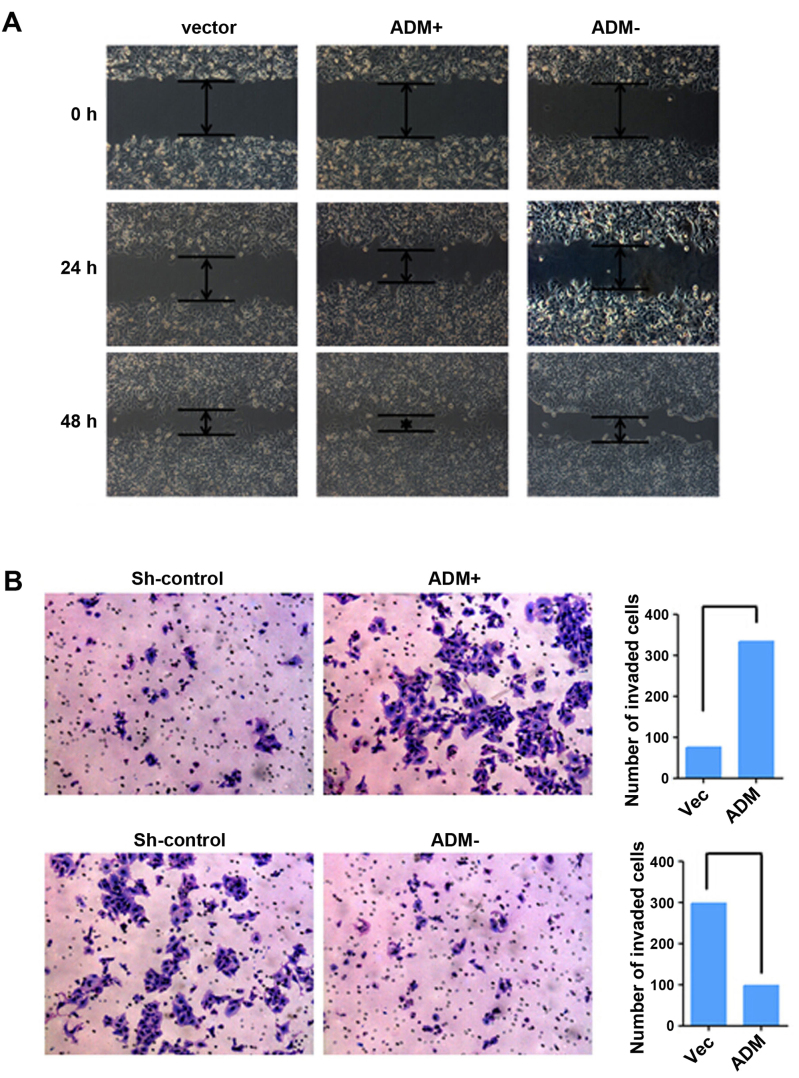
ADM promotes cell motility and ICC invasion. (A) Wound-healing assay showing that ADM promoted cell migration. Representative images were taken at 0, 24 and 48 h after scratching. (B) Transwell invasion assay showing that ADM promoted cell invasion. Representative images of invaded cells are shown in the left panel and the results are summarized in the right panel. The results are expressed as the mean ± SD of three independent experiments (^**^P<0.01, independent Student's t-test). ADM, adrenomedullin.

The roles of ADM in tumor cell migration and invasion were investigated by wound-healing and Transwell invasion assays. ADM-transfected cells achieved faster closure of the scratched 'wounds' than this rate in the control cells (Fig. 5A). Furthermore, cell motility and invasion in the Transwell invasion assays were significantly increased by ADM transfection (P<0.05, Fig. 5B). Opposite results were obtained in the ADM-silenced cells.

### Effect of ADM on epithelial-mesenchymal transition (EMT) in ICC

**Figure 6:**
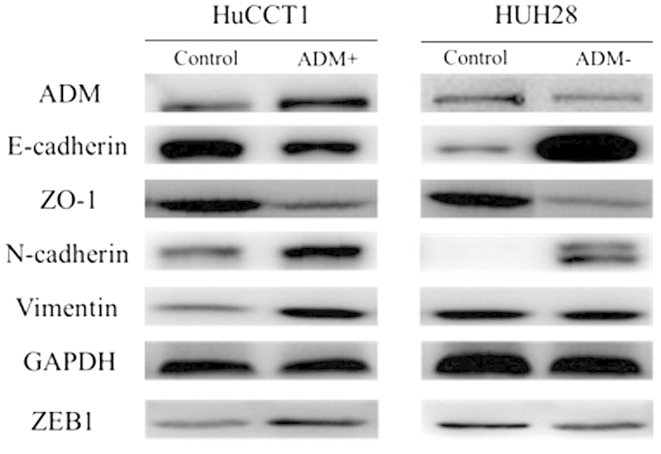
ADM activates ZEB1 to induce EMT. Western blot analyses comparing the relative expression of ADM, E-cadherin, ZO-1, N-cadherin, vimentin and ZEB1 in the ADM-expressing (ADM^+^) and ADM-silenced (ADM^−^) cells and their respective control cells. GAPDH expression was used as the loading control. ADM, adrenomedullin; EMT, epithelial-mesenchymal transition.

EMT is one of the key events in tumor invasion and metastasis. We therefore investigated the effects of ADM on EMT by analyzing the expression levels of EMT markers and EMT-related transcription factors, and by morphological changes in the ICC cell lines. Western blotting demonstrated decreased expression levels of the epithelial markers E-cadherin and ZO-1, and increased expression of the mesenchymal markers vimentin and N-cadherin, and the EMT-related transcription factors ZEB1 and ZEB2 in the ADM-transfected cells (Fig. 6), compared with the control cells. Opposite expression patterns of these genes were observed in the ADM-silenced cells.

## Discussion

ADM expression has been demonstrated in many human malignant cells (14,15), but to the best of our knowledge, the present study is the first to report ADM expression and its specific mechanisms in ICC. ADM protein was highly expressed in ICCs according to IHC, while ADM mRNA expression levels were higher in the tumor tissues compared with that in the peritumoral and healthy tissues. Overexpression of ADM was significantly associated with poorer OS (P<0.001) and tumor recurrence (P<0.01). These results suggest that ADM acts as an oncogene with an important role in ICC progression.

EMT is considered to be a critical mechanism involved in cancer metastasis (16,17); however, compared with other types of human cancers, few studies have focused on the significance of EMT in ICC (18,19). Our functional studies demonstrated that ADM had strong tumorigenicity, with its overexpression promoting cell growth, migration and invasion. These results were in agreement with those of previous studies (8,20). The two main types of primary liver cancer are HCC and ICC. Although ADM may promote metastasis in both ICC and HCC, the mechanisms appear to differ, possibly because of the different origins of the two tumors. ICC results from the malignant transformation of cholangiocytes. Gene expression profiles analyzed by microarray identified ADM as a metastasis-associated gene. Previous IHC analysis showed that HCC samples with intra-hepatic metastasis expressed strong ADM immunoreactivity in the cytoplasm of tumor cells (21). ADM signaling has been reported to be hypoxia-inducible and functionally active in HCCs (20). In the present study, morphological changes in ICC cells suggest a link between the biological function of ADM and EMT induction. As anticipated, mesenchymal markers were significantly upregulated in stable ADM transfectants, whereas epithelial markers were significantly downregulated. In addition, ADM silencing was associated with increased expression of epithelial markers and decreased levels of mesenchymal markers. ZEBl is the most important transcription factor in the regulation of EMT in epithelial cells (22,23). The present study provides the first evidence demonstrating that ADM induces EMT through ZEB1.

Our study had some limitations. First, although animal models offer an opportunity to bridge the gap between *in vitro* findings and clinical applicability, such animal experiments were not performed in the present study because of a lack of time and experimental conditions. Second, we focused mainly on patients with resectable ICC at a single institution, and further multicenter studies are needed to validate the findings.

In conclusion, overexpression of ADM in human ICC cells led to increased growth, invasion and metastasis *in vitro*. High ADM levels in clinical ICC specimens correlated with poor prognosis. These findings suggest that ADM should be evaluated as a potential novel therapeutic target in ICC with the aim of improving the currently poor outcome of this disease.

## Figures and Tables

**Table I:** Clinicopathological characteristics of the 133 patients with ICC.

Demographics	Recurrence group A (n=54)	Non-recurrence group B (n=79)	P-value^b^
Gender			
Male	28	52	0.10606
Female	26	27	
Age (years)			
>60	27	31	0.21914
≤60	27	48	
Differentiation^a^			
I–II	33	53	0.47882
III–IV	21	26	
Encapsulation			
Complete	4	10	0.33254
None	50	69	
CA19–9 (U/ml)			
≤37	28	38	0.01929
>37	42	25	
Lymph node metastasis			
Presence	11	13	0.77174
Absence	49	66	
Tumor size (cm)			
≤5	21	42	0.10539
>5	33	37	
ADM			
Low ≤6	23	50	0.01848
High	31	29	

a Tumor differentiation was assigned by Edmondson's grading system.

b P-value for the comparison of cohort B with cohort A; ADM, adre-nomedullin; CA19–9, carbohydrate antigen 19–9; ICC, intrahepatic cholangiocellular carcinoma.

**Table II:** Univariate and multivariate analyses of factors associated with survival and recurrence.

Factor	Univariate		Multivariate	
	Hazard ratio (95% CI)	P-value^a^	Hazard ratio (95% CI)	P-value^a^
Gender (female vs. male)	0.782 (0.430–1.423)	0.421		
Age (>60 vs. ≤60 years)	0.899 (0.472–1.713)	0.746		
Tumor size (>5 vs. ≤5 cm)	0.902 (0.493–1.650)	0.738		
Differentiation (I–II vs. III–IV)	**2.581** (**1.216–5.481**)	**0.014**	**2.177** (**1.016–4.665**)	**0.045**
CA19–9 (>37 vs. ≤37 U/ml)	0.779 (0.409–1.480)	0.445		
Encapsulation (complete vs. none)	**2.609** (**0.921–7.338**)	**0.041**	**1.497** (**0.503–4.456**)	**0.469**
ADM (positive vs. negative)	**2.869** (**1.453–5.665**)	**0.002**	**2.412** (**1.207–4.823**)	**0.013**

ADM, adrenomedullin; CA19–9, carbohydrate antigen 19–9.
